# A New Record of Longicorn Beetle, *Acanthophorus rugiceps*, from India as a Root Borer on Physic Nut, *Jatropha curcas*, with a Description of Life Stages, Biology, and Seasonal Dynamics

**DOI:** 10.1673/031.012.14101

**Published:** 2012-12-05

**Authors:** Mathyam Prabhakar, Y.G. Prasad, G.R. Rao, B. Venkateswarlu

**Affiliations:** Central Research Institute for Dryland Agriculture, Santoshnagar, Hyderabad, 500059, Andhra Pradesh, India.

**Keywords:** biofuel crop, cerambycid beetle, pest

## Abstract

Longicorn beetle, *Acanthophorus rugiceps* Gahan (Coleoptera: Cerambycidae), is reported for the first time as a confirmed host on physic nut, *Jatropha curcas* L. (Malpighiales: Euphorbiaceae), from India, causing extensive damage to roots. Plants of three years age and above were prone to attack by this pest. In a six year study beginning in 2005, about 11.3 percent of plants in a 16.25 acre physic nut plantation were severely damaged by *A. rugiceps*. Life stages of *A. rugiceps*, including egg, larvae, pupae, and adult, are described with a note on their habitat, biology, and behavior. Strategies to manage this pest on physic nut are discussed.

## Introduction

With the ever increasing demand for petroleum products world over, crude oil prices are expected to remain high for many years to come. Hence, several countries have intensified their search for alternate sources of fuels. Tree-borne oil seed, *Jatropha curcas* L. (Malpighiales: Euphorbiaceae), commonly known as physic nut, contains 27–40 percent oil that can be processed to produce a high quality biofuel and has attracted research attention as a source of biofuel in many countries, including India. The Planning Commission (Government of India) has identified 13.4 million ha of land, mostly from cultivable fallows through a joint forestry management scheme, and also as a hedge crop around few agricultural fields, for cultivation of *J. curcas* (GOI 2003). A national mission on biofuels was initiated during March 2004. With considerable efforts from various stakeholders, about 0.5 million ha were brought under physic nut cultivation in India (USDA 2010). With the increase in its cultivated area, a variety of new insect pests and diseases have been reported on Jatropha across India (Chitra and Dhyani 2006; Manoharan et al. 2006; Tewari et al. 2007; Prabhakar et al. 2008; Srinivasa Rao et al. 2011). However, none of these pests were observed causing plant mortality leading to eventual loss of entire plantation. Recently, longicorn beetle, *Acanthophorus rugiceps* Gahan (Coleoptera: Cerambycidae), was causing severe root damage in physic nut plantations in a research farm at Hyderabad, India. The first report of *A. rugiceps* from the Bombay presidency, India, was from an unknown host (Gahan 1906), and its range extended to Pakistan and southeastern Iran (Villiers 1967). Later, this species was reported from the Persian Gulf region with a limited description of adult behaviour (Rejzek et al. 2005). However, details on biology, behavior, description of immature stages, nature of damage, and seasonal dynamics of *A. rugiceps* are unknown. This paper provides added details on the biology of this species.

## Materials and Methods

Over the past eight years, a large collection of germplasm lines of *J. curcas* were evaluated for different traits at the Hayathnagar research farm of the Central Research Institute for Dryland Agriculture, Hyderabad, India (17° 21′ 1.483′ N, 78° 36′ 6.116″ E). Soil in the farm is a *typic haplustalf* (red sandy loam) with shallow depth (40 to 45 cm), and organic carbon content is 0.4%. Blocks of about four acres each were planted every alternate year beginning in 2003 at a spacing of 3×3 m. The crop was grown completely under normal rainfall conditions with no supplementary irrigation by following agronomic practices such as weeding, fertilizer application, and pruning (Rao et al. 2010). Fortnightly observations on pest incidence were recorded on 30 randomly selected plants per acre. Activity of the adult insects was monitored throughout the year using light traps (Deccan Tekno Corporation, Hyderabad, India). Severely infested plants that collapsed to the ground were dug out and inspected for larvae in the root zone. Biology of the larvae was studied by rearing field collected mature larvae on potted physic nut plants (25 1 capacity) maintained in a green house. Over a period of five years, a large number of eggs (n = 198), larvae of different sizes (n = 54), pupa (n = 8), and adults (n = 38) of *A. rugiceps* that mostly came from field collections were observed. Morphometric measurements of different life stages were made by processing the digital images using ProgRes Capture software v. 2.7 (Jenoptik Optical Systems, http://www.jenoptik-inc.com/). Adult beetles collected in the light traps were used for some of the behavioral studies.

## Results and Discussion

The first symptoms of root borer damage on physic nut were noticed during October 2006 in a three year old plantation, and in the subsequent years this infestation spread to other blocks. In all the fields, the first root borer infestation was observed only in three to four year old plantations. Affected plants looked healthy during the initial stages of infestation.

Severely affected plants showed sudden wilting with heavy leaf fall, eventually leading to the collapse of the entire plant at the root collar region. After excavating the base of damaged plants, creamy white larvae of various sizes were found in the root zone. Extensive tunnelling by these larvae rendered roots and parts of the main stem hollow and fragile, thereby causing collapse of the plant. At the time of this study, a total of 816 physic nut plants, distributed in four different blocks in a 16.25 acre plantation (11.3%), were infested with this root borer. This infestation spreads to new plants each year.

Pale-yellow, oval shaped, large eggs (length 6.8 ± 0.26 mm, breadth 3.46 ± 0.30 mm) weighing about 39 mg each were found on the soil near the root zone. The sites of ovipostion were easily distinguished by the scraping marks made by the adult females, and the eggs were often found covered with a thin layer of soil. Most of the eggs were found within a radius of one to five feet of the adult exit holes on the soil. Examination of surface soil where empty egg shells were found revealed neonate larvae burrowing into soil. Several eggs observed in the field hatched in three to seven days, but none of the eggs laid under laboratory conditions hatched (87 eggs from three gravid females). Larvae were smooth, soft, creamy-white, with protruding thoracic segments. The head capsule and mandibles were highly sclerotized. Size of larvae varied depending on their age; the smallest observed was 0.71 cm length and 0.55 mm breadth, while the largest was 23.04 cm length and 2.59 cm breadth. The fully grown larva weighed about 63.9 g. This may be one of the largest larvae reported in this group of insects. The number of larval instars and their duration could not be precisely determined, due to prolonged life cycle and difficulty in rearing the larvae under laboratory conditions. Surprisingly, some of the field collected larvae reared in the laboratory survived in dry soil for about 9 months, even without any food (released into soil in earthen pots without plants), and subsequently died. Pupation was found in soil two to three feet below the surface in a large oval earthen cell made up of chewed wooden material and soil particles. After successful completion of pupal period, adults emerged through exit holes at the end of the long tunnels (up to 92 cm). Presence of these circular holes near the affected plants was an indication of adult emergence, which was generally observed during May to July after the onset of monsoon showers. This was further confirmed by adults (both males and females) caught in light traps during the same period. However, the number of adults caught in these traps was low compared to a large number of emergence holes observed each year during this period.

The adult beetles were large, reddish-brown, with strong mandibles, and exhibited a pronounced sexual dimorphism. Males were larger in size (mean body length with mandibles 7.82 ± 0.35 cm, range 7.25–8.16 cm; breadth 2.19 ± 0.19 cm, range 1.9–2.28 cm) with strong mandibles and long antennae (6.08 ± 0.72 cm) compared to females (mean body length with mandibles 5.06 ± 0.53 cm, range 4.54–5.90 cm; breadth 1.88 ± 0.26 cm, range 1.5–2.16 cm; antennae length 3.65 ± 0.12 cm). Adult beetles were poor fliers, presumably due to their large size. A few dead adults were found on the ground, most likely killed by predatory ants.

However, some of the adults collected in light traps, which were set 20 m away from the main plantation at a height of six feet above ground, indicated their ability to fly short distances. The adults, particularly males, after slight disturbance, tried to attack with their strong mandibles. Males in isolation remained sluggish without much movement in the rearing containers, but became active within few minutes after release of adult females in the same container. Adult longevity was between six to 18 days under laboratory conditions, and no feeding was observed by either sex. Adults appeared to be nocturnal in habit, as they were rarely sighted in fields during the day time.

The only other species of *Acanthophorus* reported so far from India is *A. serraticornis*, which is also the largest reported beetle from the country, (Ghate et al. 2003) with *Mangifera indica, Bombax malabaricum, Morus alba*, and *Shorea robusta* serving as its hosts (Beeson 1941). *Prosopis, Acacia, Zizipus, Calotrpis*, and *Phoenix* have been reported as suspected host plants for *A. rugiceps* in the Persian Gulf region (Rejzek et al. 2005). However, this is the first report of *J. curcas* as a confirmed host for *A. rugiceps*. Some of the adult attributes of *A. rugiceps* described by Rejzek et al. 2005, such as nocturnal habitat, sexual dimorphism, and body measurements, are more or less in support of the present findings. Though the number of *A. rugiceps* adults caught in the light trap was less (28) in our study, it showed the attraction of adults towards light, which is contrary to the earlier report by Rejzek et al. (2005). However, several factors influence their catch, such as type of illumination source, light intensity, distance and height of placement of the trap, and, more importantly, body size and flight capacity of insects like *A. rugiceps*.

Some of the efforts made to recover the infested plants by soil drenching with Chlorpyriphos 20% EC, dusting soil around the main stem with Methyl parathion 2D, and applying Phorate 10G granules in the root zone did not yield desired results. This result may be due to the fact that insecticides applied on the surface of soil seldom reach the root zone at two to three feet below, where most of the larvae were found. Even spot application of Dichlorvos 76% EC by drilling a long, narrow and deep hole in the soil near the root zone of infested plants did not result in any significant control. As observed in the present study, the crucial period for control of this pest appears to be during the time of adult emergence and subsequent oviposition, which is generally after the onset of the first monsoon rains during May to July. Most of the adults emerge during this period and lay eggs within six to 15 days on the soil surface near the infested plants. This period after the adult emergence appears to be the weak link in the life cycle of this pest, so any control interventions should be targeted to kill the newly emerging adults and eggs laid on surface soil near the plant base. Once the egg hatches and the larvae burrow into the soil in the root zone, it is very difficult to kill them. Identification of sex pheromone, development of trap design, use of entomo pathogenic nematodes or fungi, identification of alternate hosts, and identification of new physic nut cultivars from the existing germplasm collection that can withstand the borer attack are some of the researchable issues that need to be addressed in order to control this pest more effectively. This study of *A. rugiceps* is based on a single location, and thus establishes susceptibility of physic nut to this pest.

**Figure 1.**
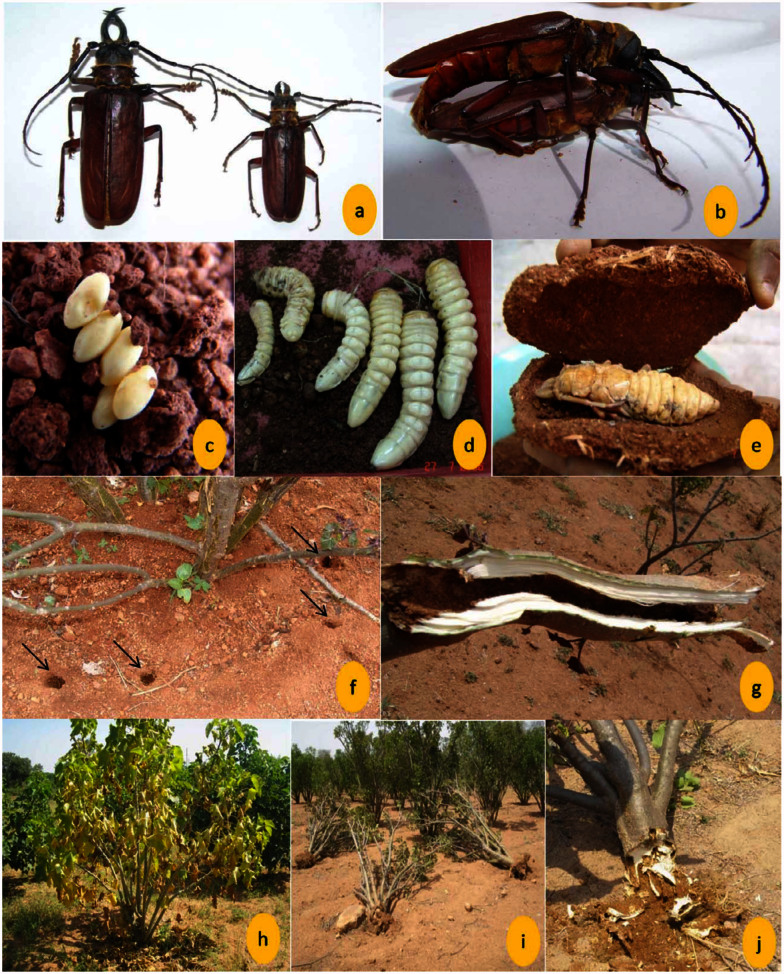
Life stages of *Acanthophorus rugiceps* and symptoms of damage on *Jatropha curcas*. a. Adult beetles, male (left), female (right); b. Copulating adults; c. Eggs; d. Larvae of different sizes; e. Pupa inside an earthen cell; f. Exit holes of adults (shown with arrows) around the plant; g. Stem tunnelling by larvae; h. Withering and leaf fall symptom; i. Dislodgement of whole plants due to borer attack; j. Extensive root damage by the larvae. High quality figures are available online.
